# Treatment with the dual-incretin agonist DA-CH5 demonstrates potent therapeutic effect in a rat model of Wolfram Syndrome

**DOI:** 10.3389/fendo.2023.1234925

**Published:** 2023-10-13

**Authors:** Toomas Jagomäe, Nayana Gaur, Kadri Seppa, Riin Reimets, Marko Pastak, Mihkel Plaas, Allen Kaasik, Eero Vasar, Mario Plaas

**Affiliations:** ^1^ Laboratory Animal Centre, Institute of Biomedicine and Translational Medicine, University of Tartu, Tartu, Estonia; ^2^ Eye Clinic of Tartu University Hospital, Tartu, Estonia; ^3^ Ear Clinic of Tartu University Hospital, Tartu, Estonia; ^4^ Department of Pharmacology, Institute of Biomedicine and Translational Medicine, University of Tartu, Tartu, Estonia; ^5^ Department of Physiology, Institute of Biomedicine and Translational Medicine, University of Tartu, Tartu, Estonia

**Keywords:** wolfram (DIDMOAD) syndrome, incretin mimetics, glucose intolerance, dual incretins, animal models - rodent, Wfs1

## Abstract

**Aim:**

Wolfram Syndrome (WS) is a rare condition caused by mutations in *Wfs1*, with a poor prognosis and no cure. Mono-agonists targeting the incretin glucagon-like-peptide 1 (GLP-1) have demonstrated disease-modifying potential in pre-clinical and clinical settings. Dual agonists that target GLP-1 and glucose-dependent insulinotropic polypeptide (GIP-1) are reportedly more efficacious; hence, we evaluated the therapeutic potential of dual incretin agonism in a loss-of-function rat model of WS.

**Methods:**

Eight-month-old *Wfs1* knock-out (KO) and wild-type control rats were continuously treated with either the dual agonist DA-CH5 or saline for four months. Glycemic profile, visual acuity and hearing sensitivity were longitudinally monitored pre-treatment, and then at 10.5 and 12 months. Pancreata and retina were harvested for immunohistological analysis.

**Results:**

DA-CH5 therapy reversed glucose intolerance in KO rats and provided lasting anti-diabetogenic protection. Treatment also reversed intra-islet alterations, including reduced endocrine islet area and β-cell density, indicating its regenerative potential. Although no rescue effect was noted for hearing loss, visual acuity and retinal ganglion cell density were better preserved in DA-CH5-treated rats.

**Conclusion:**

We present preclinical evidence for the pleiotropic therapeutic effects of long-term dual incretin agonist treatment; effects were seen despite treatment beginning after symptom-onset, indicating reversal of disease progression. Dual incretins represent a promising therapeutic avenue for WS patients.

## 1 Introduction

Mutations in the *WFS1* gene encoding the Wolframin1 protein (WFS1) cause the classical form of the rare condition Wolfram Syndrome (WS). WFS1 plays critical roles in regulating endoplasmic reticulum (ER) stress, calcium homeostasis and mitochondrial function and is broadly expressed across several organs (1). It can also directly modulate major physiological systems like the Renin-Angiotensin-Aldosterone and Kinin-Kallikrein systems (RAAS, KKS) that regulate critical functions, including blood pressure, fluid balance, and inflammatory response (1–3). WS is therefore a multi-systemic condition and hallmarks include diabetes, optic nerve atrophy, deafness and progressive neurodegeneration. Consequently, prognoses are extremely poor with a median lifespan of 30-40 years and effective disease modifying treatments are urgently needed (4). Drug repurposing efforts have identified various molecular pathways, like the ER stress response and calcium signaling, for pharmacological intervention (5). For instance, chemical chaperones like valproate and dantrolene have demonstrated protective effects in animal models of WS and are now being explored in clinical trials (6–9). The prototypic Sigma1 receptor agonist PRE-084 and AMX0035, a combination of two chaperones (4-phenylbutyric acid and tauroursodeoxycholic acid, Amylyx Pharmaceuticals) also provided symptomatic benefit in pre-clinical models (10, 11). Notably, AMX0035, although originally approved for the treatment of amyotrophic lateral sclerosis in the USA, is now being evaluated in a Phase II clinical trial with WS patients (12).

Drugs targeting the metabolic incretin system have proven particularly efficacious; the incretins are hormones secreted by the gut in response to nutrient ingestion (13). They include glucagon-like peptide-1 (GLP-1) and glucose-dependent insulinotropic polypeptide (GIP), each of which have specific receptors. Incretins regulate glycemic homeostasis via their additive actions on insulin and glucagon and also provide trophic support to pancreatic beta cells (14, 15). Incretin mimetics like GLP-1 receptor (GLP-1R) agonists (for e.g., Liraglutide, Semaglutide), were initially developed for the treatment of Type 2 diabetes mellitus (T2DM) and obesity but have demonstrated therapeutic benefit across several domains in WS. For instance, our group has extensively tested Liraglutide (LIRA) in an established loss-of-function rat model of WS (*Wfs1* coding exon 5 knock-out, *Wfs1*KO) to show that treatment can substantially delay the onset of glucose intolerance and loss of vision (16–18). Therapeutic benefits were also evident in aged animals where treatment began after symptom onset; KO rats displayed improved performance on cognitive tasks and had reduced ER stress, neuroinflammation, optic nerve atrophy and retinal ganglion cell death (18, 19). Similarly, the long-lasting agonist dulaglutide had both a preventive and curative effect on glucose intolerance in a mouse model of WS (20).

We have additionally shown that 16-month long preventive LIRA treatment commencing at 2 months of age was not only well-tolerated, but also conferred significant protection against the progression of hyperglycaemia and vision loss in *Wfs1*KO rats (16). Consequently, GLP-1 agonists are already being used by the majority of paediatric WS patients for symptom management (21–23).

While the underlying basis of incretin agonism’s therapeutic effects remain to be fully elucidated, several—likely overlapping—mechanisms of action have been reported. These include promoting proliferation and repair, protecting against oxidative stress and excitoxicity-induced apoptosis (24–26), and regulating cellular processes like inflammation, ER stress and autophagy (27, 28). For instance, the GLP-1R agonist exenatide protected iPSC-derived *WFS1*-deficient beta cells and cerebellar neurons from ER stress and mitigated mitochondrial dysfunction and the production of reactive oxygen species (20). Similarly, while *Wfs1* deficiency can directly impair components of the autophagy lysosomal pathway (29), GLP-1R agonists have been shown to restore lysosomal function (30, 31).

Incretin agonism may also drive the “resensitization” of peripheral and central insulinogenic signaling pathways and a resultant decrease in pro-inflammatory factors (32). This would also explain the treatment-induced benefits observed in conditions like Alzheimer’s and Parkinson’s Disease (AD, PD), as impaired cerebral metabolism and insulin responsiveness have been implicated in several neurodegenerative pathologies (33).

In lieu of this, reports of highly potent dual GLP-1/GIP agonists are relevant, given that these also appear to be better tolerated by patients (34). These are unimolecular peptides that provide targeted and balanced agonism of both the GLP-1 and GIP receptors, thereby addressing both insulin resistance and deficiency (35). Their synergistic pharmacology has yielded superior metabolic outcomes in rodent and non-human primate models and human trials of obesity and T2DM than selective GLP-1 mono-therapy (36). Dual agonists have also demonstrated stronger therapeutic potential in neurodegenerative disorders (37–39); in a mouse model of PD, the dual agonist DA-CH5 had better blood-brain barrier (BBB) penetration than a comparable LIRA dose and reduced glial activation and apoptotic signaling to a greater extent (38). Similarly, another dual agonist DA-JC4, attenuated memory impairments and levels of phosphorylated tau protein aggregates in a rat model of AD (35).

Accordingly, we assessed the efficacy of long-term dual agonist treatment in a rat model of WS and show that dual agonists have pleotropic therapeutic effects and represent a new class of potential disease-modifying therapies for WS.

## 2 Materials and methods

### 2.1 Animals

Experiments were approved by the Estonian Project Authorization Committee for Animal Experiments (No 204, 11th November 2019), and performed in accordance with the ARRIVE guidelines and European Communities Directive of September 2010 (2010/63/EU). Generation and phenotyping of Wfs1 mutant rats has been previously described (*Wfs1* coding exon 5 knock-out, *Wfs1* KO) (40). Breeding and genotyping were conducted at the Laboratory Animal Centre at the University of Tartu, Estonia. We used male homozygous *Wfs1* KO rats and Wild-type (WT) littermates as controls. Animals were housed in cages (2-4/cage) under a 12-hour light/dark cycle (lights on at 7 a.m.) with maintenance at dim light conditions (60–80 lux) to minimize retinal phototoxicity. Animals had ad-libitum access to food and water except during active experiments and were weighed weekly (Sniff universal mouse and rat maintenance diet (Sniff #V1534) and reverse osmosis-purified water). All experiments were performed between 9 a.m. and 5 p.m. Experimenters were blinded to genotype during all bio-and immunohistochemical analyses.

### 2.2 Intraperitoneal glucose tolerance tests and clinical chemistry

Animals were deprived of food for 3 hours prior to testing, but had continued access to water. Glucose (Sigma-Aldrich) was dissolved in a 0.9% saline solution (20% w/v) and administered intraperitoneally (i.p.) at a dose of 2 g/kg. Blood glucose was measured at: 0 min (before administration), 30 min, 60 min, 120 min and 180 min after administration from the tail vein using a hand-held glucometer (Accu-Check Go, Roche, Mannheim, Germany). Tail vein blood samples taken immediately prior to and 30 min after glucose administration were retained for analyses. Samples were allowed to clot (30 min, room temperature), centrifuged (2000 x g, 15 min, 4°C), and the serum was collected and stored at -80°C. Commercially validated sandwich ELISA kits were used in accordance with manufacturer instructions (Crystal Chem): Insulin (cat# 90060) and C-peptide (cat # 90055). Absorbance was measured at 450 nm (wavelength correction 620 nm) and concentrations were calculated with 4-parameter logistic regression analysis.

“Habituation” IPGTTs were performed prior to all experiments (acute and long-term) to control for anxiety-induced fluctuations. Following this, animals received subcutaneous (s.c.) saline injections for 7 consecutive days to accustom them to experimenter handling. All IPGTTs (pre-treatment baseline and follow-ups) were always performed 24 hours after the last drug/saline injection. **Supplementary Figure 1** illustrates the IPGTT timetable.

### 2.3 Drugs and drug administration

Amino acid sequences for the dual-incretin agonists DA-JC4 and DA-CH5 were obtained from Feng et al. (39) and synthesized by Pepscan; purity was > 95% as determined by HPLC and UPLC-MS. Peptides were pre-weighed and dry stored at -80°C; fresh stock solutions were prepared in PBS immediately prior to injection. Stock solutions were diluted 1:10 in saline. Liraglutide (Victoza®, Novo Nordisk Medical, Denmark) was also prepared fresh in saline. All drugs were administered by daily s.c. injection (injection volume = 1 ml/kg, all injections between 8:00-11:00 am.).

#### 2.3.1 Acute treatment and screening paradigm

Five-month-old *Wfs1* KO and WT rats were used to examine the effects of acute treatment with selected single- and dual-incretin agonists (timeline in **Supplementary Figure 1**) and identify the most promising candidates for independent long-term treatment. After the baseline IPGTT, animals were randomly allocated to one of the experimental groups below:

i) Saline (SAL) (WT n = 10, *Wfs1* KO n = 8) 0.9% solution.
ii) DA-CH5 (WT n = 8, *Wfs1* KO n = 9) 25 nmol/kg.
iii) DA-JC4 (WT n = 8, *Wfs1* KO n = 8) 25 nmol/kg.
iv) Liraglutide (LIRA) (WT n = 8, *Wfs1* KO n = 7) 25 nmol/kg.

Dosing for DA-CH5 and DA-JC4 was determined from the literature, as these concentrations were neuroprotective in a mouse model of PD (38, 39). Liraglutide was administered at an equimolar dose to allow direct comparison. Treatment was administered for 7 consecutive days (daily, s.c.) followed by a final IPGTT.

#### 2.3.2 Long-term treatment with the dual incretin agonist DA-CH5

Eight-month-old *Wfs1* KO and WT rats were used for the long-term treatment paradigm; tested compounds were selected based on the results of the acute screening paradigm (timeline in **Supplementary Figure 1**). After the baseline IPGTT, animals were randomly allocated to one of the experimental groups below:

i. SAL (WT n = 9, *Wfs1* KO n = 10) 0.9% solution.

ii. DA-CH5 (WT n = 8, *Wfs1 KO* n = 10) 25 nmol/kg.

Treatments were administered for 4 months (daily, s.c.), with follow-up IPGTTs at 10.5 and 12 months. Rats received weekly weigh-ins and monthly base blood sugar level monitoring. Supportive insulin treatment (100 IU/ml, Levemir, Novo Nordisk, Denmark) was initiated in hyperglycemic *Wfs1* KO rats; animals with a basal blood glucose level of 10 mmol/l received 1 IU/kg of insulin (twice daily, s.c.). Every subsequent 5 mmol/l increase in blood glucose corresponded to a 1 IU/kg increase in the insulin dose (for e.g., blood glucose 20 mmol/l = 6 IU/kg insulin, twice daily).

### 2.4 Visual acuity estimation and cataract scoring

Visual acuity was evaluated as part of the long-term treatment paradigm at the ages of 8, 10.5 and 12 months using a virtual optomotor task (OptoMotry, Cerebral Mechanics Inc., Alberta, Canada) as previously described (18). Briefly, animals were placed in the center of a virtual rotating cylinder displaying vertical bars and their tracking behavior was recorded. Clockwise and anti-clockwise rotations were detected by the left and right eye, respectively. Data are presented as the mean of clockwise and anti-clockwise recording.

Structural lens changes were evaluated at the ages of 8 and 12 months using a portable slit lamp (after dilation with 1% tropicamide); cataract severity was assessed in a blinded fashion by an ophthalmologist as previously described (16, 41).

### 2.5 Hearing evaluation

Hearing sensitivity was assessed as previously described (16); briefly, a clinical screening instrument (Sentiero Diagnostics, PATH medical GmbH, Germering, Germany) with a frequency modulated distortion product otoacoustic emission (DPOAEs) tympanometry upgrade (FMDPOAETM) was used to measure otoacoustic emissions. Measurements were recorded at the ages of 8, 10.5 and 12 months. Prior to all measurements, the ears were visually inspected to rule out alternative causes for conductive hearing loss (cerumen, inflammation, etc.).

### 2.6 Tissue collection

Animals were weighed and anesthetized with an intraperitoneal injection of ketamine/dexmedetomidine solution (ketamine 150 mg/kg and dexmedetomidine 0.5 mg/kg), injection volume of 0.1 ml/100 g of body weight). Following confirmation of reflex loss, the intraperitoneal cavity and thorax were opened, and blood samples were taken from the left ventricle using BD Vacutainer® PrecisionGlide™ single-used needles (18g x 1.5″, Cat # 360748) and collected in VACUETTE® serum separator tubes. Thereafter, animals were transcardially perfused with a pre-flush of 0.1 M phosphate buffer (PB) followed by 4% paraformaldehyde (PFA) in PB. Tissues of interest were carefully dissected and weighed (brain, pancreas, m. tibialis anterior) and immersion fixed in 4% PFA/phosphate buffered saline (PBS) overnight at 4°C. The superior poles of the eyes were marked by punctuation to allow orientation of the retinas. Retinas from the left eye were dissected and processed as previously described (42). Samples were rinsed several times with PBS and impregnated in a 30% sucrose/PBS solution for 2-3 days at 4°C. Tissues were frozen at -80°C until further immunostaining; all immunostaining details are provided in **Table 1**.

**Table 1:** Antibody information for immunostaining experiments.

Target	Primary Antibody (Catalog number, dilution, duration)	Secondary Antibody (Catalog number, dilution, duration)
Insulin	PA1-26938, 1:100, 72 hours at 4°C	111-545-045, 1:500, 45 min at RT
Glucagon	03-16032, 1:500, 72 hours at 4°C	106-025-006, 1:500, 45 min at RT
GLP-1	ABS 033-04-02, 1:500, 72 hours at 4°C	706-025-148, 1:500, 45 min at RT
Somatostatin	ab111912, 1:500, 72 hours at 4°C	A32728, 1:500, 45 min at RT
Brn3A	411004, 1:500, 72 hours at 4°C	106-545-008, 1:500, 45 min at RT
GAP-43	ab107159, 1:1000, 72 hours at 4°C	A32728, 1:500, 45 min at RT

GAP-43, growth-associated protein 43; GLP-1, Glucagon-like peptide 1; RT, room temperature.

### 2.7 Endocrine islet analyses

Endocrine islet mass determination was performed as previously described (43). Briefly, pancreas sections were immunostained for islet cell populations; α- (Glucagon+), β-(Insulin+) and δ-cell (SST+); and GLP-1 as an indicator for overall islet health. Sections were imaged with a 10x Objective (HC PL FLUOTAR 10x/0.32) mounted to an Aperio VERSA 10 Brightfield, Fluorescence & FISH Digital Pathology Scanner (Leica Biosystems). Acquired images were analyzed using Aperio ImageScope Software v12.4.3.500 (Leica Biosystems). Total pancreas area was estimated using Positive Pixel Count FL v1 algorithm (image zoom: 0.1; minimal intensity threshold: 0.05-0.07). Islets were identified based on positive immunostaining and the mass was estimated as follows: (total islet area/total pancreas area) * pancreas mass.

Individual cell populations (Glucagon+ α cells, Insulin+ β cells, SST+ δ cells, GLP+ cells) were assessed in eight individual islets per animal. Sections were imaged using a 20x Objective (HC PL FLUOTAR 20x/0.55 DRY) with at least 10 focal planes (z-stacks, plane interval = 2 µm) captured per islet. Subsequent analyses were performed using the Visiopharm (2021.09) software suite, with at least 25% of the total area analyzed per islet. Positively stained cells (α, β, δ) were manually counted within randomly generated counting frames (counting frame area 5%); only cells that had a visible nucleus were counted. Average section thickness was 14 µm as determined manually.

### 2.8 Retinal morphometry and flat-mount immunohistochemistry

Prior to sectioning, the right eyes (n = 5-7/group) were immersed in Tissue-Tek™ O.C.T. Compound (Thermo Fischer Scientific) and the superior-inferior axis was set perpendicular to the blade. Twenty µm thick sections were collected at the level of the optic nerve head. Retinal thickness measurements were recorded from sections where the optic nerve head was visible at an interval of 500 µm. Retinas from the left eye (n = 5-6/group) were subjected to flat-mount immunohistochemistry as previously described (42). Briefly, retinal ganglion cells (RGCs) were visualized using immunofluorescent labelling of the transcription factor Brn3a (44). Analyses were performed on four retinal quadrants in relation to the optic nerve head: superior-proximal and superior-distal, inferior-proximal and inferior-distal regions. Brn3a+ cells in each region were manually counted within 16 randomly generated counting frames (ImageJ 1.53n Unbiased Counting Frame software plugin v 1.0; total frame area = 2.022 mm^2^).

### 2.9 Statistical analysis and data visualization

Statistical analyses and data visualization were performed using the SPSS (IBM, v29) and GraphPad Prism (v 9.4.1) software packages for Windows. Data normality was assessed using the Shapiro Wilk Test. Initial three-way ANOVAs showed no significant interaction between time *x* genotype *x* treatment for any parameters. Therefore, simple main effects were investigated using either two-way mixed or independent ANOVAs.

Serum biochemical assays were assessed using multiple paired t-tests. All analyses were corrected for multiple comparisons using the Bonferroni method (ANOVA-based analyses) or the Holm-Sidak method (Paired t-test analyses). Two-sided statistical significance was set at p < 0.05. Data are presented with the mean and standard error of the mean (SEM) unless otherwise stated.

The GNU Image Manipulation Program (GIMP, version 2.10.24) was used for microscopy image processing.

## 3 Results

### 3.1 Acute DA-CH5 therapy improves glucose intolerance and acts as a secretagogue in symptomatic *Wfs1* KO rats

Five-month-old *Wfs1* KO rats and matched WT controls were acutely treated to assess the efficacy of the dual-incretin agonists DA-JC4 and DA-CH5 relative to the established mono-agonist LIRA. This age was chosen as this is when KO animals clearly exhibit glucose intolerance and the anti-diabetogenic properties of selected compounds can therefore be tested (17, 40).

**Figure 1:**
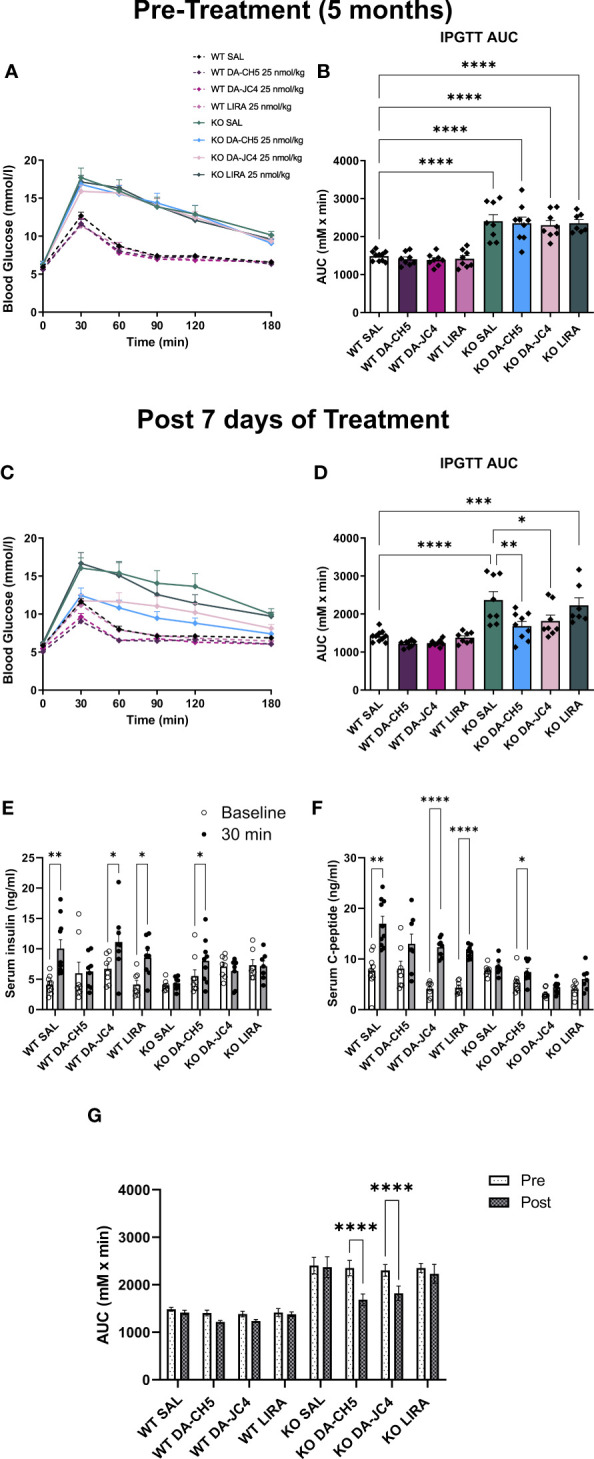
Acute dual-incretin agonist treatment reverses manifest glucose intolerance in *Wfs1* KO rats. Five-month-old wild-type and *Wfs1* KO rats were treated with either saline (SAL), the dual-incretin agonist DA-CH5 (25 nmol/kg), the dual-incretin agonist DA-JC4 (25 nmol/kg), or the GLP-1 mono-agonist Liraglutide (LIRA, 25 nmol/kg) to assess the effects of acute therapy on glucose intolerance. **(A–D)** Blood glucose levels as assessed by intraperitoneal glucose tolerance test (IPGTT) and calculated area under the curve (AUC). Tests were performed **(A, B)** prior to and **(C, D)** after 7 days of treatment. Glucose-stimulated **(E)** serum insulin, and **(F)** C-peptide secretion profile after the treatment period. **(G)** Comparative overview of pre-post treatment AUC changes. Data in **(B, D, E–G)** are presented as the mean and standard error of the mean. Symbols represent individual mice. **(B, D, G)** Two-way analysis of variance. **(E, F)** Multiple paired t-tests with Holm-Sidak correction for multiple comparisons. * *p* < 0.05, ** *p* < 0.01, *** *p* < 0.001, **** p < 0.0001, n = 7-9/group.

Pre-treatment IPGTT and AUC comparisons indicated that while fasting blood sugar was similar between genotypes, KO animals across all groups were hyperglycemic immediately following glucose administration (**Figures 1A, B**). *Wfs1* KO rats were therefore already glucose intolerant, which is in line with previous phenotypic observations (17). Following a 7-day treatment period, both DA-CH5- and DA-JC4-treated KO rats were normoglycemic, as no significant AUC differences were noted relative to WT rats (**Figures 1C, D**); in contrast, LIRA-treated KO rats remained glucose intolerant. DA-CH5-treated rats also displayed a noticeable reduction in AUC relative to SAL-treated KO rats, indicating significant glycemic normalization. DA-CH5 also had a potent secretagogue effect: amongst KO rats, only those treated with DA-CH5 had a significantly improved serum insulin and c-peptide response to glucose load (**Figures 1E, F**).

In summary, acute therapy with the dual GLP-1/GIP agonists DA-CH5 and DA-JC4 normalized the dysglycemic phenotype in *Wfs1* KO rats to a greater extent than equimolar treatment with the mono-agonist liraglutide, as evidenced by pre- vs. post-treatment AUC comparisons (**Figure 1G**). Of the two dual agonists, DA-CH5 had a stronger insulinogenic effect and was therefore selected for further long-term experiments.

### 3.2 Long-term DA-CH5 therapy confers enduring anti-diabetogenic protection to older symptomatic *Wfs1* KO rats

We then wanted to examine the effects of long-term treatment with DA-CH5 in an independent cohort. Eight-month-old *Wfs1* KO rats and matched WT controls were continuously administered either DA-CH5 or SAL over four months, with experimental follow-ups at 10.5 and 12 months. This age was selected as this is when deficits in sensory function, including vision and hearing, develop (16, 17, 40). Furthermore, we wanted to examine whether dual incretin therapy is protective even if treatment commences at an advanced disease stage, given that human WS patients often experience a significant diagnostic delay: diagnoses are typically confirmed after optic nerve atrophy has already occurred (1).

**Figure 2:**
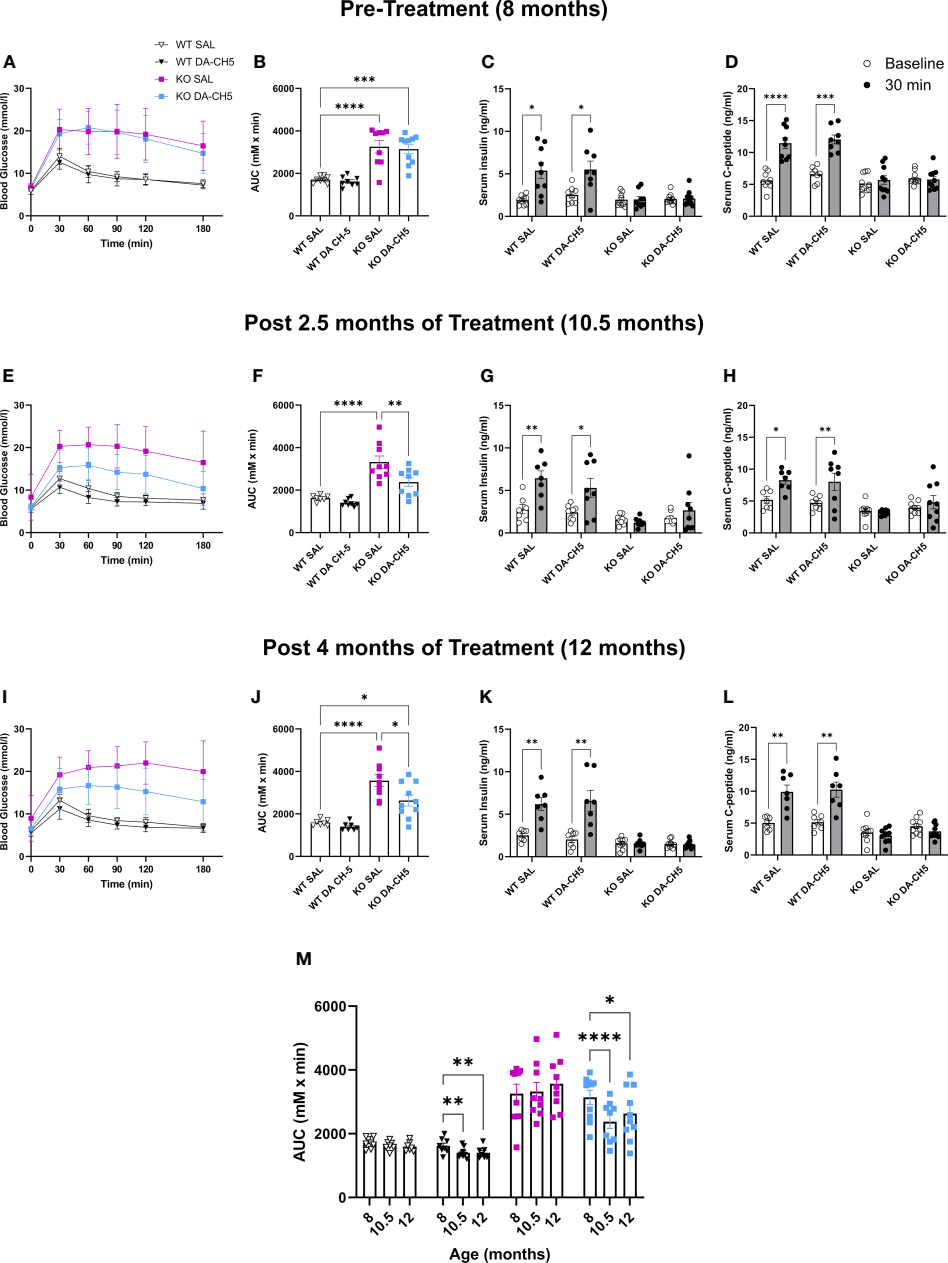
Long-term dual-incretin agonist treatment stably reverses glucose intolerance in older *Wfs1* KO rats. Eight-month-old wild-type and *Wfs1* KO rats were continuously treated with either saline (SAL) or the dual-incretin agonist DA-CH5 (25 nmol/kg) for four months with longitudinal experimental monitoring at: **(A–D)** pre-treatment 8 months, **(E–H)** 10.5, and **(I–L)** 12 months of age. **(A, B, E, F, I, J)** Blood glucose levels as assessed by intraperitoneal glucose tolerance test (IPGTT) and calculated area under the curve. **(C, D, G, H, K, L)** Glucose-stimulated **(C, G, K)** serum insulin and **(D, H, L)** C-peptide secretion profile. **(M)** Longitudinal within-group AUC changes. Data are presented as the mean and standard error of the mean. Symbols represent individual mice. **(F, J, M)** Two-way analysis of variance. **(G, H, K, L)** Multiple paired t-tests with Holm-Sidak correction for multiple comparisons. * *p* < 0.05, ** *p* < 0.01, *** *p* < 0.001, **** p < 0.0001, n = 8-10/group.

The pre-treatment IPGTT at 8 months confirmed that *Wfs1* KO rats in both the SAL and DA-CH5 groups were severely glucose intolerant (**Figures 2A, B**) and that this intolerance was more pronounced than in younger five-month-old KO rats, indicating phenotypic progression (5 month vs. 8 month mean AUC = 2353 vs. 3198, [F (3, 97) = 82.31, *p < 0.0001*]). Hormone secretion was also significantly impaired, as no increase in either serum insulin or c-peptide levels were noted following glucose administration (**Figures 2C, D**). As expected, SAL-treated KO animals were also glucose intolerant at the 10.5-month timepoint (**Figures 2E, F**); in contrast, DA-CH5 treated KO rats displayed a significant improvement in glycemic phenotype as their AUC was significantly lower than that of SAL-treated KO rats (**Figure 2F**). Furthermore, DA-CH5 therapy normalized dysglycemia in KO rats to the extent that no significant differences in the AUC were noted relative to WT rats. Curiously, and in contrast to the results observed in the acute therapy paradigm, no concomitant improvement was noted in glucose-stimulated insulin (**Figure 2G**) and c-peptide levels (**Figure 2H**); KO rats in the SAL and DA-CH5 groups had similar secretory profiles. The final follow-up at the age of 12 months revealed that the AUC in DA-CH5-treated KO rats was still significantly lower than in SAL-treated KO rats, indicating longitudinal maintenance of glucose tolerance (**Figures 2I, J**). As with the previous 10.5-month timepoint, no significant changes were noted in hormone secretion in response to glucose load (**Figures 2K, L**).

In summary, long-term DA-CH5 therapy was able to reverse manifest diabetes and glucose intolerance even in older rats with more pronounced symptoms and this therapeutic effect was maintained even as animals aged (**Figure 2M**).

### 3.3 Long-term DA-CH5 therapy rescues disrupted endocrine islet composition and morphology in symptomatic *Wfs1* KO rats

**Figure 3:**
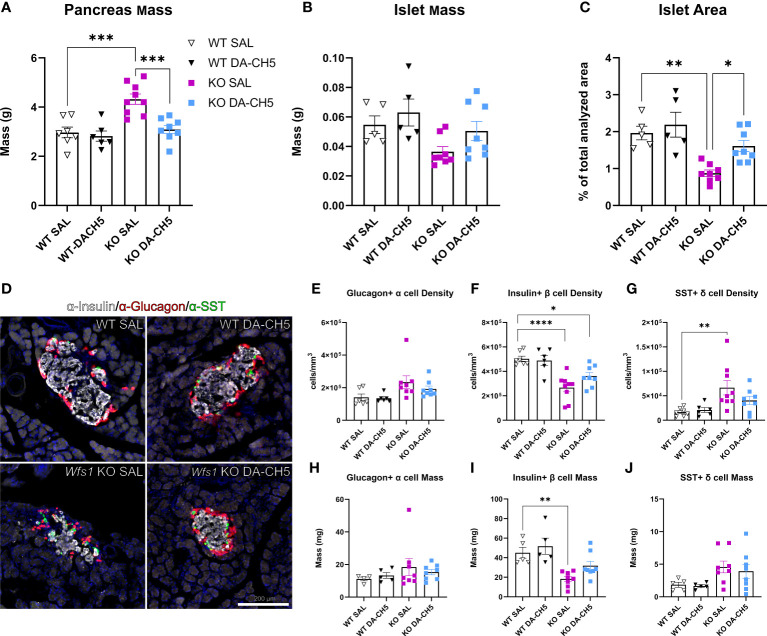
Long-term dual-incretin agonist treatment rescues altered pancreatic and endocrine islet architecture in *Wfs1* KO rats. Eight-month-old wild-type (WT) and *Wfs1* KO rats were continuously treated with either saline (SAL) or the dual-incretin agonist DA-CH5 (25 nmol/kg) for four months, following which pancreata were harvested and histologically examined. Between-group differences in **(A)** pancreatic mass, **(B)** endocrine islet mass, and **(C)** islet area were examined; islet area was calculated as a percentage of the total analysed pancreatic area. **(D)** Representative confocal images of pancreatic sections immunostained for insulin (red), Glucagon, (grey), and somatostatin (SST, green) to demarcate endocrine cell populations: Glucagon+ alpha (α) cells, Insulin+ beta (β) cells, and SST+ delta (δ) cells. Between-group differences in **(E–G)** cell density and **(H–J)** mass were quantified for individual populations. Data are presented as the mean and standard error of the mean. Symbols represent individual mice. Two-way analysis of variance. * *p* < 0.05, ** *p* < 0.01, *** *p* < 0.001, **** p < 0.0001, (a, n = 8-10/group; b-j, n = 5-8/group). Scale bar, 250 µm.

We next wanted to examine whether the glycemic improvement observed with DA-CH5 therapy also translated to changes in the pancreatic cytoarchitecture. For this, pancreata were harvested from all animals at the last timepoint (12 months) and subjected to immunohistological analysis. Gross inspection revealed that pancreata from SAL-treated KO rats were significantly enlarged relative to SAL-treated WT rats (**Figure 3A**), while endocrine islet mass and area were reduced (**Figures 3B, C**). Closer inspection of the islet also reflected several morphological aberrations, including severely compromised islet organization (**Figure 3D**). While islets from WT rats displayed a characteristic circular core of β (insulin+) cells surrounded by a mantle of α (Glucagon+) and δ cells (SST+), no clear organization was visible in islets from KO rats. Rather, islets were irregularly shaped, with no distinct core/mantle separation and random distribution of all constituent cell types. Quantitative analyses confirmed that islets from KO rats also had significant compositional irregularities (**Figures 3E–G**); a twofold decrease was noted for β cell density, while conversely, a fourfold increase was noted for δ cell density. Glucagon+ α cell density was also increased, albeit non-significantly. These irregularities were also reflected in calculated masses albeit not to the same extent (**Figures 3H–J**). Interestingly, staining for GLP-1 also confirmed poor overall islet health in KO rats: GLP-1+ cell density was significantly increased and cells were randomly dispersed across the entire islet cross-section (**Supplementary Figure 3**).

Crucially, DA-CH5 therapy substantially corrected these alterations; both gross pancreatic and islet mass were normalized, with DA-CH5-treated KO rats even showing significantly increased islet area relative to SAL-treated rats (**Figures 3A–C**). Islets also appeared larger with at least partial restoration of morphology and cell distribution (**Figure 3D**). Individual cell densities were maintained to the extent that no significant differences in α and δ cells were noted relative to WT rats (**Figure 3I**); β cell density reduction relative to WT rats was also not as pronounced as it was in the KO-SAL group (DA-CH5 KO ≈32% reduction vs. SAL-KO ≈61% reduction, **Figure 3J**). DA-CH5 therapy also normalized the increased GLP-1+ cell density observed in KO rats.

In summary, *Wfs1* KO rats developed profound gross pancreatic and intra-islet irregularities that were significantly reversed by long-term DA-CH5 therapy.

### 3.4 Wolfram Syndrome-associated visual acuity loss is attenuated by long-term DA-CH5 therapy

**Figure 4:**
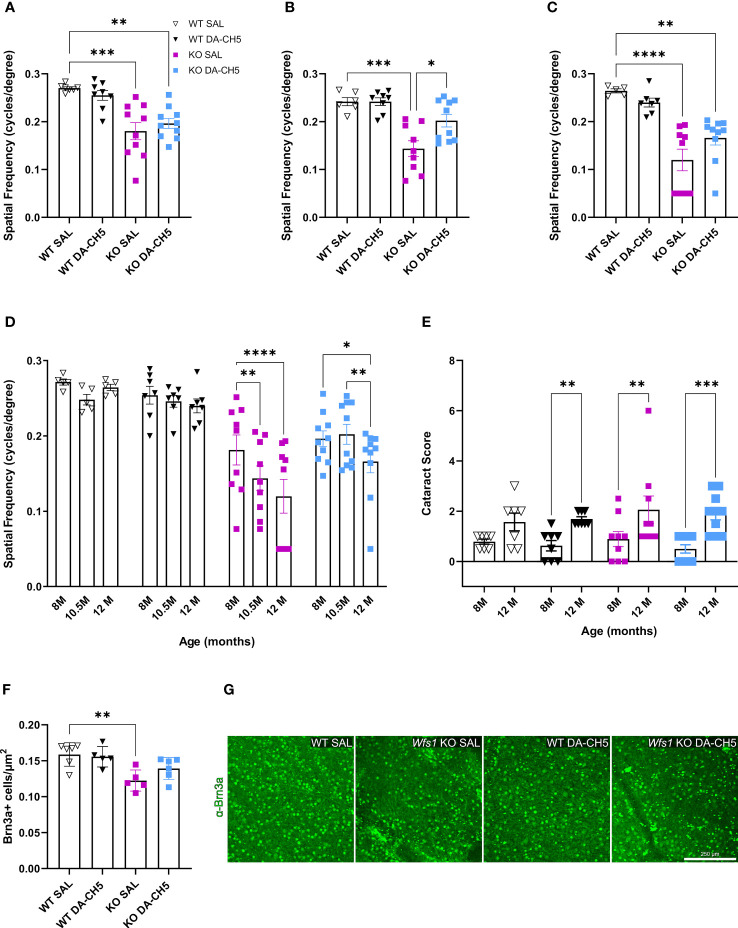
Visual acuity and retinal ganglion cell density are better preserved in *Wfs1* KO rats receiving long-term dual-incretin agonist treatment. Eight-month-old wild-type (WT) and *Wfs1* KO rats were continuously treated with either saline (SAL) or the dual-incretin agonist DA-CH5 (25 nmol/kg) for four months. **(A–C)** Visual acuity as measured by the optokinetic reflex response from both eyes at: **(A)** pre-treatment 8 months, **(B)** 10.5, and **(C)** 12 months of age. **(D)** Longitudinal within-group changes in visual acuity. **(E)** Cataract severity progression across the experimental period. **(F)** Quantification of Brn3a+ retinal ganglion cells from retina harvested at experimental endpoint (12 months). **(G)** Representative confocal images of retinal flat mounts immunostained for Brn3a (green, retinal ganglion cells). Data are presented as the mean and standard error of the mean. Symbols represent individual mice. Two-way analysis of variance. * *p* < 0.05, ** *p* < 0.01, *** *p* < 0.001, (a-e, n = 8-10/group; f, n = 5-7/group). Scale bar, 250 µm.

Since ophthalmologic deficits are a key feature of WS (30, 45–47), we investigated whether DA-CH5’s protective effects extend beyond glycemic control. Visual acuity, as measured by optokinetic response, was longitudinally monitored. *Wfs1* KO rats had significantly impaired visual acuity relative to WT rats at the 8-month timepoint, as also previously reported (**Figure 4A**). However, the 10.5-month follow-up indicated treatment-induced differences: acuity in DA-CH5-treated KO rats was significantly improved relative to SAL-treated KO rats and restored to the extent that no differences were noted relative to WT rats (**Figure 4B**). Although a slight deterioration was noted at 12 months, visual acuity was still better preserved in DA-CH5 treated KO rats (**Figures 4C, D**). Of note, cataract severity was monitored to ensure it didn’t influence any of the observed between-group differences. Scores uniformly declined across all groups between the ages of 8 and 12 months (**Figure 4E**), with no significant between-group differences, indicating that while DA-CH5 treatment did not have a discernible effect on cataract development and progression, it drove functional improvements in vision.

RGC density was examined using the established marker Brn3a to see if changes corroborated functional improvements in visual acuity (44). Comparisons across the whole retina revealed both genotype- and treatment-associated differences; Brn3a+ cell density was significantly lower in SAL-treated KO rats relative to WT rats, confirming the vulnerability of this population in WS (**Figures 4F, G**). Regional analyses indicated that this difference was most evident in the superior distal area (**Supplementary Figure 4C**). Surprisingly, DA-CH5 exerted a neuroprotective effect, as no significant differences in density were noted between treated KO and WT rats (**Figure 4G**). To examine if DA-CH5 also had regenerative potential, we examined growth-associated protein-43 (GAP-43) expression in RGC axons. GAP-43 plays a role in neural injury response and regeneration and is required for axonal growth and elongation (48). Visual inspection revealed no differences in expression among the four groups; localization patterns were also similar, with robust staining noted in the inner plexiform layer extending to the ganglion cell layer, and gradually diminishing towards the optic nerve head (**Supplementary Figure 4D**).

Finally, retinal thickness along was measured along the vertical meridian, as alterations have previously been reported in human patients (45). However, no genotype- or treatment-associated differences were noted (**Supplementary Figure 4G**).

In summary, progressive deficits in vision over the WS disease course were attenuated by DA-CH5 therapy, as evidenced by functional improvements and preserved RGC density.

### 3.5 Long-term DA-CH5 therapy does not rescue sensorineural hearing loss in symptomatic *Wfs1* KO rats

As progressive hearing loss is a clinical hallmark of WS, we longitudinally tracked hearing sensitivity using DPOAEs (49–51). In line with previous reports, sensitivity was already significantly compromised in KO animals at the age of 8 months (**Supplementary Figure 5A**) (16). DA-CH5 therapy was unable to reverse this as SAL- and DA-CH5-treated KO rats displayed similar profiles at subsequent follow ups (**Supplementary Figures 5B, C**). Finally, sensitivity did not progressively deteriorate in KO rats and remained relatively stable until the 12-month endpoint.

## 4 Discussion

While no cure currently exists for the progressive condition WS, incretin mimetics have emerged as a promising therapeutic option. Studies have primarily reported on mono-agonists targeting the GLP-1 receptor, as these have demonstrated robust anti-obesogenic and -hyperglycaemic effects in both pre-clinical and clinical settings. However, increased efficacy and patient compliance are limited by gastrointestinal complications associated with higher doses (52).

Accordingly, we show here that long-term dual-incretin agonist treatment demonstrated significant therapeutic effect across multiple phenotypic domains in a rat model of WS. To begin with, results from our acute treatment paradigm confirmed previous reports of the enhanced efficacy of dual agonists relative to mono-agonists (36, 53, 54). Both of the tested dual agonists DA-CH5 and DA-JC4, were able to normalize glucose intolerance and glucose-stimulated secretory response in symptomatic 5-month-old *Wfs1* KO rats. Conversely, treatment with an equimolar dose of the mono-agonist Liraglutide only induced weight loss (**Supplementary Figure 2B**). Previous studies using this model have shown that an almost four-fold higher dose is needed for Liraglutide to mediate its anti-diabetic and neuroprotective effects, further underscoring the superior potency of dual agonists (16, 18, 19). This is particularly relevant given that longitudinal studies have indicated significant variability in treatment response to mono-agonists: for instance, approximately 50% of *Wfs1* KO rats developed insulin-dependent diabetes despite lifelong Liraglutide treatment that commenced in the pre-symptomatic phase (16). The increased potency of dual-incretin agonists like DA-CH5 may derive from them being more physiologically appropriate and harnessing the complementary actions of GLP-1 and GIP. Although hyperglycaemia can severely impair GIP’s insulinotropic effects (55), these can be restored by using GLP-1’s preserved insulinotropic and glucagonostatic effects to normalize blood sugar. GIP receptor agonism can then induce insulin secretion by 1) direct β cell stimulation, 2) potentiating glucagon release and 3) triggering paracrine α-β cell communication, thereby delivering a stronger final synergistic effect (56–59). Increased efficacy is also driven by the differential action of dual agonists on GLP-1 and GIP receptor trafficking and recycling (58, 59).

Crucially, the same therapeutic benefit was noted in our long-term treatment paradigm despite treatment commencing when animals were 8 months old i.e., further along the disease course. This is particularly relevant given our previous observations that Liraglutide treatment was unable to rescue overt glucose intolerance in older *Wfs1* KO rats (16, 18, 19). Continuous DA-CH5 therapy reversed manifest glucose intolerance in KO animals and maintained normoglycemia as they aged, underscoring the superior efficacy of dual agonists. Intriguingly, and in contrast to the acute treatment paradigm, no significant changes were noted in serum insulin and c-peptide levels; SAL- and DA-CH5-treated KO rats had similar glucose-stimulated secretory profiles at both the 10.5- and 12-month timepoints. However, the C-peptide index (C-peptide: blood glucose at 30 min following glucose administration) significantly improved in treated KO animals, particularly at the 10.5-month timepoint, indicating the presence of functional insulin. DA-CH5-associated glycaemic improvements may therefore at least partially be driven by improved β-cell function and/or insulin processing, particularly since WFS1 plays a critical role in proinsulin (insulin precursor) folding and processing (60). For instance, Finan et al. reported that diabetic rats treated with a dual agonist produced less insulin in response to glycemic challenge than those treated with the mono-agonist liraglutide, indicating an improvement in insulin sensitivity (36).

Alternatively, reduced insulin secretion may simply be the result of a prolonged normoglycemic state and decreased β cell stimulation as suggested by Irwin et. al., who reported a similar phenomenon in obese mice. Sub-chronic co-administration of GIP and GLP-1 analogues progressively reduced non-fasting plasma glucose levels but had no effect on plasma insulin (61).

The benefits of DA-CH5 treatment were also evident at the tissue level, as therapy normalized the increased pancreatic mass seen in KO rats. We speculate that this increase may have been driven by the exocrine pancreas, as it comprises most of the organ (~90% of mass) (62). Furthermore, we observed no evidence of pathological hyperplasia, oedema, or connective tissue proliferation. Disruptions in the exocrine pancreas may also underlie the frequent gastrointestinal complaints by WS patients (1), as proper digestive function and nutrient ingestion are tightly regulated by crosstalk between the exocrine pancreas and small intestine. Interestingly, Morikawa et al. also recently reported significant hypervascularization and immune cell infiltration in the pancreatic islets of *Wfs1* KO mice, which may also contribute to increased tissue mass (63, 64).

Closer analysis of the endocrine islet revealed that DA-CH5 treatment significantly increased occupied area, indicating its curative potential. Of note, unlike % area, islet mass may not fully reflect the magnitude of the degenerative process, as it is calculated using pancreas mass (43). Values may therefore be skewed if pancreata are significantly heavier as in the KO-SAL group. Treatment also normalized intra-islet morphological and compositional alterations; notably, alterations were observed across all constituent cell types. Insulin+ β cell density was significantly reduced, while Glucagon+ α and SST+ δ cell densities were increased in SAL-treated KO rats. Gorgogetias et al. also reported reduced β cell and increased α cell densities in a mouse model of WS, confirming that the intricate regulatory feedback loops between endocrine hormones are markedly disturbed in WS and there is significant intra-islet stress (20). This was supported by our observation of significantly increased GLP-1+ cell density in islets from KO rats; similar alterations have been previously reported in animal models of and human patients with T2DM. For instance, Campbell et al. reported that islets from donors with T2DM had significantly more GLP-1+ α cells (65). Indeed, while physiological α-cell expression of GLP-1 has been reported in both murine and human islets, expression is increased under conditions of metabolic stress (65–69).

In WS, a cascade of events may therefore converge on the final insulin-dependent diabetic phenotype seen in patients; the loss of functional WFS1 and associated ER stress eventually leads to β-cell failure, compromised insulin synthesis and the breach of a “pro” to “anti-regenerative” glucose threshold (70). While α and δ cell densities may initially increase to help maintain glucose homeostasis, these increases, coupled with already low insulin levels, contribute to hyperglucagonemia. Ultimately, persistent and uncontrolled hyperglycaemia and associated cellular stress prevent β-cell recovery and compensatory proliferation, culminating in a permanently disturbed metabolic microenvironment. By reducing glycaemic load, DA-CH5 therapy may therefore give β cells a chance to regenerate, as seen here: β cell density and mass in KO rats were increased following DA-CH5 treatment. Treatment can improve insulin sensitivity and thereby lessen the “secretory burden” on β cells, enable the proliferation of surviving cells, and also encourage γ-aminobutyric acid (GABA)-induced trans differentiation of α to β-like cells (36, 71). Incretin agonism, as with DA-CH5, can also directly stimulate GABA formation, which itself has anti-inflammatory, anti-apoptotic and insulinogenic effects (72–77).

DA-CH5 treatment also conferred protection against visual impairments, as acuity progressively declined in SAL-treated KO rats, while DA-CH5-treated rats showed a significant improvement at 10.5 months. Although there was a deterioration at 12 months, this may have been influenced by cataract progression. Behavioural assessments of visual acuity were supplemented by immunohistological analysis of Brn3a+ RGC density; however, assessing vision is challenging given species-specific differences in retinal organization. For instance, rodents do not have a well-defined fovea and rod-cone distribution follows a characteristic dorsal-ventral gradient across the retina. However, these differences were accounted for by performing density analyses across the whole retina and regional quadrants (78). As with the optometry task, RGC density was indeed better preserved in DA-CH5 treated KO rats, confirming that therapy was protective. Regional analyses indicated that density reduction in KO rats was particularly pronounced in the superior-distal quadrant (**Supplementary Figure 4C**); intriguingly, this regional specificity was also observed for NeuN+ cells in an independent cohort of 3-month-old animals (**Supplementary Figure 4B**). Although not statistically significant, the result suggests that vision loss in WS may begin relatively early and set the stage for further degeneration. Indeed, electrophysiological retinal impairments were detectable by the age of 4 months in a mouse model of WS (79). The same study reported that RGC loss follows secondary to myelin thinning and axonal degeneration within the optic nerve; the same sequence has been noted in our rat model and explains the progressive worsening on functional assessments of visual acuity (16, 18). Finally, DA-CH5 therapy had no effect on WS-associated hearing loss; cochlear hearing levels were impaired in KO rats at 8 months as previously observed, and remained stable in both SAL and DA-CH5 treatment groups (16). As with vision however, progressive hearing loss is difficult to model in animals, given that it is also variable in human patients; onset can range from birth to adolescence (16, 80, 81). Early diagnosis and intervention are therefore crucial to prevent or at least mitigate irreversible damage.

The limitations of the present study include the lack of information on insulin sensitivity as an insulin tolerance test was not performed. Additionally, the long-term paradigm did not include a treatment-arm with the GLP-1 mono-agonist Liraglutide. As previously mentioned, this was because our acute paradigm revealed that at an equimolar dose, liraglutide yielded no glycemic normalization. This precluded a direct comparison with DA-CH5 in the subsequent longitudinal study. Future studies may therefore consider including independent treatment-arms with GLP-1 and GIP mono-agonists. An additional point to consider is that DA-CH5 is not structurally identical to commercially retailed dual agonists being considered for clinical trials with WS patients e.g., Tirzepatide (82). Unfortunately, we were unable to include these in the present study as these were not available for exploratory pre-clinical testing.

To conclude, the work presented is the first to demonstrate the superior therapeutic benefit of dual incretin agonists via meaningful improvements in various *in vivo* outcomes in a rat model of WS. To illustrate, while our previous longitudinal studies have shown that long-term Liraglutide treatment has pleiotropic effects, we show here that the same effects can be achieved with a much lower dose of DA-CH5. Furthermore, while the therapeutic protection conferred against vision was comparable, DA-CH5 had superior efficacy in normalizing WS-associated glucose intolerance, even in older animals with advanced disease. This has implications for a broad spectrum of metabolic disorders, particularly since dual agonism can prevent glycaemic volatility and safeguard against hypoglycaemia via GIP’s differential action on α cells (83). Dual agonists like DA-CH5 have also demonstrated superior neuroprotection in models of neurodegenerative diseases because they have been specifically designed to optimize BBB penetration (35, 38, 84). Accordingly, the data presented here suggest that dual incretin agonism may be an effective therapeutic strategy for patients with WS, particularly given the potent rescue effects observed for glucose intolerance and visual acuity. Further, commercially available candidates like Tirzepatide, owing to their pharmacokinetic properties, are associated with fewer gastrointestinal effects and need to be injected less frequently, which likely increases patient compliance. Long-term clinical trials that also allow monitoring of the impact of dual agonists on neuro-ophthalmologic outcomes in WS patients are therefore warranted.

Our data additionally emphasize the need for early diagnosis to allow intervention within a suitable window. Finally, our results highlight the translational value of validated animal models for rapidly identifying potential disease-modifying compounds and longitudinally tracking their effects. We recommend that future studies investigate the protective effects of prophylactic treatment with dual incretin agonists.

## Data availability statement

The raw data supporting the conclusions of this article will be made available by the authors, without undue reservation.

## Ethics statement

The animal study was approved by Estonian Project Authorization Committee for Animal Experiments (No 204, 11th November 2019). The study was conducted in accordance with the local legislation and institutional requirements.

## Author contributions

Study conception and design: TJ, NG, EV, MaPl. Data acquisition and analysis: TJ, NG, KS, RR, MaPa, MaPl. Manuscript original draft compilation, subsequent editing, and figure preparation: TJ, NG, KS, RR, MaPa, MiPl, AK, EV, MaPl. All authors contributed to the article and approved the submitted version.

## Supplementary material

Supplementary Figure 1 Experimental timeline. Schematic representation of experimental protocols in acute and long-term treatment paradigms.

Supplementary Figure 2 Additional metabolic readouts following acute and long-term dual-incretin agonist treatment. Metabolic read-outs after **(A, B)** acute and (c-f) chronic treatment paradigms. **(A, B)** Five-month-old wild-type (WT) and *Wfs1* knock-out (KO) rats were treated with either saline (SAL), the dual-incretin agonist DA-CH5 (25 nmol/kg), the dual-incretin agonist DA-JC4 (25 nmol/kg), or the GLP-1 mono-agonist Liraglutide (LIRA, 25 nmol/kg). **(A)** C-peptide index, calculated as ratio of serum C-peptide levels to blood glucose levels 30 minutes after intraperitoneal glucose administration, and **(B)** treatment-induced changes in body weight. **(C-F)** Eight-month-old wild-type (WT) and *Wfs1* KO rats were continuously treated with either saline (SAL) or the dual-incretin agonist DA-CH5 (25 nmol/kg) for four months. **(C-E)** C-peptide index at **(C)** pre-treatment 8 months, **(D)** 10.5, and **(E)** 12 months of age. **(F)** Longitudinal changes in body weight. Data are presented as the mean and standard error of the mean. Symbols represent individual mice. Two-way analysis of variance. * *p* < 0.05, ** *p* < 0.01, *** *p* < 0.001, (a-b, n = 7-9/group; c-f, n = 8-10/group).

Supplementary Figure 3 Endocrine islet immunostaining for Glucagon-like Peptide 1. Eight-month-old wild-type (WT) and *Wfs1* KO rats were continuously treated with either saline (SAL) or the dual-incretin agonist DA-CH5 (25 nmol/kg) for four months, following which pancreata were harvested and histologically examined. **(A)** Representative confocal images of endocrine islets immunostained for insulin (red), GLP-1 (grey), and somatostatin (SST, green). **(B, C)** GLP-1+ cell density and cell mass were quantified to evaluate between-group differences. Data are presented as the mean and standard error of the mean. Symbols represent individual mice. Two-way analysis of variance. * *p* < 0.05, ** *p* < 0.01, *** *p* < 0.001*, **** p* < 0.0001, (n = 5-8/group). Scale bar, 250 µm.

Supplementary Figure 4 Additional retinal morphometric analyses. **(A, B)** NeuN+ retinal ganglion cell density across **(A)** the whole retina and **(B)** regional quadrants in 3-month-old wild-type (WT) and *Wfs1* knock-out (KO) rats. Regional quadrants were determined in relation to the optic nerve and included: superior proximal and distal and inferior proximal and distal regions. **(C)** Brn3a+ retinal ganglion cell density across regional quadrants in 8-month-old wild-type (WT) and *Wfs1* KO rats continuously treated with either saline (SAL) or the dual-incretin agonist DA-CH5 (25 nmol/kg) for four months. **(D-H)** Retinal thickness analyses in 8-month-old wild-type (WT) and *Wfs1* KO rats continuously treated with either saline (SAL) or the dual-incretin agonist DA-CH5 (25 nmol/kg) for four months. Retinal thickness was measured along the vertical meridian in 500 µm intervals from the optic nerve head. Negative and positive values represent distances from optic nerve rim (dorsal and ventral retinal planes, respectively). **(D)** Representative epifluorescent images of retinas stained for GAP-43 (green) and counterstained for H33258 (blue). **(E)** Spidergram analysis of mean retinal thickness values from the dorsal-distal (-5000 to -2500 µm), dorsal-proximal (-2500 to 0 µm), ventral-proximal (0 to 2500 µm) and ventral-distal regions (2500 to 5000 µm). **(F)** Mean retinal area. **(G, H)** Retinal thickness across **(G)** whole retina and **(H)** regional quadrants defined in **(E)**. Data are presented as the mean and standard error of the mean. Symbols represent individual mice. Two-way analysis of variance. * *p* < 0.05, ** *p* < 0.01, *** *p* < 0.001, (a-b, n = 5/group; c, n = 5-6/group; e-h, n = 5-7/group). c, cornea; eom, extraocular muscle; l, lens; on, optic nerve; r, retina; sc, sclera. Scale bar, 2.5 mm.

Supplementary Figure 5 Long-term dual-incretin agonist treatment does not mitigate hearing loss in *Wfs1* KO rats. **(A-C)** Eight-month-old wild-type (WT) and *Wfs1* KO rats were continuously treated with either saline (SAL) or the dual-incretin agonist DA-CH5 (25 nmol/kg) for four months. Cochlear hearing levels were estimated by otoacoustic emissions at **(A)** pre-treatment 8 months, **(B)** 10.5, and **(C)** 12 months of age. Data are presented as the mean and standard error of the mean. Two-way analysis of variance (n = 8-10/group). # WT SAL vs. KO SAL at *p* < 0.05, ## WT SAL vs. KO SAL at *p* < 0.01, ### WT SAL vs. KO SAL at *p* < 0.001. * WT SAL vs. KO DA-CH5 at *p* < 0.05, ** WT SAL vs. KO DA-CH5 at *p* < 0.01, *** WT SAL vs. KO DA-CH5 at *p* < 0.001.

## References

[1] Urano F. Wolfram syndrome: diagnosis, management, and treatment. Curr Diab Rep (2016) 16(1):6. doi: 10.1007/s11892-015-0702-6 26742931 PMC4705145

[2] Punapart M Seppa K Jagomäe T Liiv M Reimets R Kirillov S. The expression of RAAS key receptors, agtr2 and bdkrb1, is downregulated at an early stage in a rat model of wolfram syndrome. Genes (Basel) (2021) 12(11):1717. doi: 10.3390/genes12111717 34828323 PMC8621801

[3] Punapart M Reimets R Seppa K Kirillov S Gaur N Eskla K-L. Chronic stress alters hippocampal renin-angiotensin-aldosterone system component expression in an aged rat model of wolfram syndrome. Genes (Basel) (2023) 14(4). doi: 10.3390/genes14040827 PMC10137641 37107585

[4] Pallotta MT Tascini G Crispoldi R Orabona C Mondanelli G Grohmann U. Wolfram syndrome, a rare neurodegenerative disease: from pathogenesis to future treatment perspectives. J Transl Med (2019) 17(1):238. doi: 10.1186/s12967-019-1993-1 31337416 PMC6651977

[5] Iafusco D Zanfardino A Piscopo A Curto S Troncone A Chianese A. Metabolic treatment of wolfram syndrome. Int J Environ Res Public Health (2022) 19(5):2755. doi: 10.3390/ijerph19052755 35270448 PMC8910219

[6] Efficacy and safety trial of sodium valproate, in paediatric and adult patients with wolfram syndrome. Available at: https://clinicaltrials.gov/ct2/show/NCT03717909 (Accessed May 19, 2023).

[7] Efficacy study of daily administration of VPA in patients affected by wolfram syndrome (AUDIOWOLF) (2023). Available at: https://clinicaltrials.gov/ct2/show/NCT04940572 (Accessed May 19, 2023).

[8] A clinical trial of dantrolene sodium in pediatric and adult patients with wolfram syndrome (2023). Available at: https://clinicaltrials.gov/ct2/show/NCT02829268 (Accessed May 19, 2023).

[9] Abreu D Stone SI Pearson TS Bucelli RC Simpson AN Hurst S. A phase 1b/2a clinical trial of dantrolene sodium in patients with Wolfram syndrome. JCI Insight (2021) 6(15):1–13. doi: 10.1172/jci.insight.145188 PMC8410026 34185708

[10] Kitamura RA Maxwell KG Ye W Kries K Brown CM Augsornworawat P. Multidimensional analysis and therapeutic development using patient iPSC–derived disease models of Wolfram syndrome. JCI Insight (2022) 7(18). doi: 10.1172/jci.insight.156549 PMC9675478 36134655

[11] Crouzier L Danese A Yasui Y Richard EM Liévens JC Patergnani S. Activation of the sigma-1 receptor chaperone alleviates symptoms of Wolfram syndrome in preclinical models. Sci Transl Med (2022) 14(631):1–35. doi: 10.1126/scitranslmed.abh3763 PMC9516885 35138910

[12] AMX0035 in adult patients with wolfram syndrome. Available at: https://clinicaltrials.gov/ct2/show/NCT05676034 (Accessed May 19, 2023).

[13] Holst JJ. The incretin system in healthy humans: The role of GIP and GLP-1. Metabolism (2019) 96:46–55. doi: 10.1016/j.metabol.2019.04.014 31029770

[14] Fehmann H-C Göke R Göke B. Cell and molecular biology of the incretin hormones glucagon-like peptide-I and glucose-dependent insulin releasing polypeptide. Endocr Rev (1995) 16(3):390–410. doi: 10.1210/edrv-16-3-390 7671853

[15] Truümper A Trümper K Trusheim H Arnold R Göke B Hörsch D. Glucose-dependent insulinotropic polypeptide is a growth factor for β (INS-1) cells by pleiotropic signaling,”. Mol Endocrinol (2001) 15(9):1559–70. doi: 10.1210/mend.15.9.0688 11518806

[16] Jagomäe T Seppa K Reimets R Pastak M Plaas M Hickey MA. Early intervention and lifelong treatment with GLP1 receptor agonist liraglutide in a wolfram syndrome rat model with an emphasis on visual neurodegeneration, sensorineural hearing loss and diabetic phenotype. Cells (2021) 10(11). doi: 10.3390/cells10113193 PMC8623088 34831417

[17] Toots M Seppa K Jagomäe T Koppel T Pallase M Heinla I. Preventive treatment with liraglutide protects against development of glucose intolerance in a rat model of Wolfram syndrome. Sci Rep (2018) 8(1):1–10. doi: 10.1038/s41598-018-28314-z 29976929 PMC6033861

[18] Seppa K Jagomäe T Kukker KG Reimets R Pastak M Vasar E. Liraglutide, 7,8-DHF and their co-treatment prevents loss of vision and cognitive decline in a Wolfram syndrome rat model. Sci Rep (2021) 11(1):1–14. doi: 10.1038/s41598-021-81768-6 33500541 PMC7838169

[19] Seppa K Toots M Reimets R Jagomäe T Koppel T Pallase M. GLP-1 receptor agonist liraglutide has a neuroprotective effect on an aged rat model of Wolfram syndrome. Sci Rep (2019) 9(1):1–13. doi: 10.1038/s41598-019-52295-2 31673100 PMC6823542

[20] Gorgogietas V Rajaei B Heeyoung C Santacreu BJ Marín-Cañas S Salpea P. GLP-1R agonists demonstrate potential to treat Wolfram syndrome in human preclinical models. Diabetologia (2023) 66(7):1306–21. doi: 10.1007/s00125-023-05905-8 PMC10244297 36995380

[21] Frontino G Raouf T Canarutto D Tirelli E Di Tonno R Rigamonti A. Case report: off-label liraglutide use in children with wolfram syndrome type 1: extensive characterization of four patients. Front Pediatr (2021) 9:755365(December). doi: 10.3389/fped.2021.755365 34970515 PMC8712700

[22] Scully KJ Wolfsdorf JI. Efficacy of GLP-1 agonist therapy in autosomal dominant WFS1-related disorder: A case report. Horm Res Paediatr (2020) 93(6):409–14. doi: 10.1159/000510852 33075784

[23] Kondo M Tanabe K Amo-Shiinoki K Hatanaka M Morii T Takahashi H. Activation of GLP-1 receptor signalling alleviates cellular stresses and improves beta cell function in a mouse model of Wolfram syndrome. Diabetologia (2018) 61(10):2189–201. doi: 10.1007/s00125-018-4679-y 30054673

[24] Tamura K Minami K Kudo M Iemoto K Takahashi H Seino S. Liraglutide improves pancreatic beta cell mass and function in alloxan-induced diabetic mice. PloS One (2015) 10(5):1–15. doi: 10.1371/journal.pone.0126003 PMC4418765 25938469

[25] Deng C Cao J Han J Li J Li Z Shi N. Liraglutide activates the nrf2/HO-1 antioxidant pathway and protects brain nerve cells against cerebral ischemia in diabetic rats. Comput Intell Neurosci (2018) 2018:1–7. doi: 10.1155/2018/3094504 PMC5829331 29623090

[26] Perry T Lahiri DK Sambamurti K Chen D Mattson MP Egan JM. Glucagon-like peptide-1 decreases endogenous amyloid-β peptide (Aβ) levels and protects hippocampal neurons from death induced by Aβ and iron. J Neurosci Res (2003) 72(5):603–12. doi: 10.1002/jnr.10611 12749025

[27] Panagaki T Michael M Hölscher C. Liraglutide restores chronic ER stress, autophagy impairments and apoptotic signalling in SH-SY5Y cells. Sci Rep (2017) 7(1):16158. doi: 10.1038/s41598-017-16488-x 29170452 PMC5700973

[28] Zobel EH Ripa RS von Scholten BJ Rotbain Curovic V Kjaer A Hansen TW. Effect of liraglutide on expression of inflammatory genes in type 2 diabetes. Sci Rep (2021) 11(1):18522. doi: 10.1038/s41598-021-97967-0 34535716 PMC8448739

[29] Chen S Acosta D Li L Liang J Chang Y Wang C. Wolframin is a novel regulator of tau pathology and neurodegeneration. Acta Neuropathol (2022) 143(5):547–69. doi: 10.1007/s00401-022-02417-4 PMC13262681 35389045

[30] Zmyslowska A Waszczykowska A Baranska D Stawiski K Borowiec M Jurowski P. Four cases of Wolfram syndrome: ophthalmologic findings and complications. Sci Rep (2018) 9(3):75–80. doi: 10.1016/j.ejphar.2020.173443

[31] Cheng CK Luo J-Y Lau CW Cho WC Ng CF Ma RCW. A GLP-1 analog lowers ER stress and enhances protein folding to ameliorate homocysteine-induced endothelial dysfunction. Acta Pharmacol Sin (2021) 42(10):1598–609. doi: 10.1038/s41401-020-00589-x PMC8463564 33495519

[32] Bendotti G Montefusco L Lunati ME Usuelli V Pastore I Lazzaroni E. “The anti-inflammatory and immunological properties of GLP-1 Receptor Agonists. Pharmacol Res (2022) 182(June):106320. doi: 10.1016/j.phrs.2022.106320 35738455

[33] Kellar D Craft S. Brain insulin resistance in Alzheimer’s disease and related disorders: mechanisms and therapeutic approaches. Lancet Neurol (2020) 19(9):758–66. doi: 10.1016/S1474-4422(20)30231-3 PMC9661919 32730766

[34] Frias JP Nauck MA Van J Kutner ME Cui X Benson C. Efficacy and tolerability of tirzepatide, a dual glucose-dependent insulinotropic peptide and glucagon-like peptide-1 receptor agonist in patients with type 2 diabetes: A 12-week, randomized, double-blind, placebo-controlled study to evaluate different do. Diabetes Obes Metab (2020) 22(6):938–46. doi: 10.1111/dom.13979 PMC7318331 31984598

[35] Shi L Zhang Z Li L Hölscher C. A novel dual GLP-1/GIP receptor agonist alleviates cognitive decline by re-sensitizing insulin signaling in the Alzheimer icv. STZ rat model. Behav Brain Res (2017) 327:65–74. doi: 10.1016/j.bbr.2017.03.032 28342971

[36] Finan B Ma T Ottaway N Müller TD Habegger KM Heppner KM. Unimolecular dual incretins maximize metabolic benefits in rodents, monkeys, and humans. Sci Transl Med (2013) 5(209). doi: 10.1126/scitranslmed.3007218 24174327

[37] Hölscher C. Novel dual GLP-1/GIP receptor agonists show neuroprotective effects in Alzheimer’s and Parkinson’s disease models. Neuropharmacology (2018) 136:251–9. doi: 10.1016/j.neuropharm.2018.01.040 29402504

[38] Zhang L Zhang L Li Y Li L Melchiorsen JU Rosenkilde M. The novel dual GLP-1/GIP receptor agonist DA-CH5 is superior to single GLP-1 receptor agonists in the MPTP model of parkinson’s disease. J Parkinsons Dis (2020) 10:523–42. doi: 10.3233/JPD-191768 31958096

[39] Feng P Zhang X Li D Ji C Yuan Z Wang R. Two novel dual GLP-1/GIP receptor agonists are neuroprotective in the MPTP mouse model of Parkinson’s disease. Neuropharmacology (2018) 133:385–94. doi: 10.1016/j.neuropharm.2018.02.012 29462693

[40] Plaas M Seppa K Reimets R Jagomäe T Toots M Koppel T. Wfs1-deficient rats develop primary symptoms of Wolfram syndrome: Insulin-dependent diabetes, optic nerve atrophy and medullary degeneration. Sci Rep (2017) 7(1):1–16. doi: 10.1038/s41598-017-09392-x 28860598 PMC5579261

[41] Livesey JC Wiens LW von Seggern DJ Barlow WE Arnold A. Inhibition of radiation cataractogenesis by WR-77913. Radiat Res (2014) 141(1):99–104. 7997522

[42] Geeraerts E Dekeyster E Gaublomme D Salinas-Navarro M De Groef L Moons L. A freely available semi-automated method for quantifying retinal ganglion cells in entire retinal flatmounts. Exp Eye Res (2016) 147:105–13. doi: 10.1016/j.exer.2016.04.010 27107795

[43] Iglesias J Barg S Vallois D Lahiri S Roger C Yessoufou A. PPARβ/δ affects pancreatic β cell mass and insulin secretion in mice. J Clin Invest (2012) 122(11):4105–17. doi: 10.1172/JCI42127 PMC3484427 23093780

[44] Nadal-Nicolás FM Jiménez-López M Sobrado-Calvo P Nieto-López L Cánovas-Martinez I Salinas-Navarro M. Brn3a as a marker of retinal ganglion cells: Qualitative and quantitative time course studies in naïve and optic nerve-injured retinas. Investig Ophthalmol Vis Sci (2009) 50(8):3860–8. doi: 10.1167/iovs.08-3267 19264888

[45] Hoekel J Narayanan A Rutlin J Lugar H Al-Lozi A Hershey T. Visual pathway function and structure in Wolfram syndrome: Patient age, variation and progression. BMJ Open Ophthalmol (2018) 3(1):1–6. doi: 10.1136/bmjophth-2017-000081 PMC5895968 29657975

[46] Hoekel J Chisholm SA Al-Lozi A Hershey T Earhart G Hullar T. Ophthalmologic correlates of disease severity in children and adolescents with Wolfram syndrome. J AAPOS (2014) 18(5):461–465.e1. doi: 10.1016/j.jaapos.2014.07.162 25439303 PMC4476046

[47] Seynaeve H Vermeiren A Leys A Dralands L. Four cases of Wolfram syndrome: ophthalmologic findings and complications. Bull Soc Belge Ophtalmol (1994) 252:75–80. 7894760

[48] Chung D Shum A Caraveo G. GAP-43 and BASP1 in axon regeneration: implications for the treatment of neurodegenerative diseases. Front Cell Dev Biol (2020) 8:567537(September). doi: 10.3389/fcell.2020.567537 33015061 PMC7494789

[49] Genís D Dávalos A Molins A Ferrer I. Wolfram syndrome: A neuropathological study. Acta Neuropathol (1997) 93(4):426–9. doi: 10.1007/s004010050635 9113209

[50] Hilson JB Merchant SN Adams JC Joseph JT. Wolfram syndrome: A clinicopathologic correlation. Acta Neuropathol (2009) 118(3):415–28. doi: 10.1007/s00401-009-0546-8 PMC2758421 19449020

[51] Karzon R Narayanan A Chen L Lieu JEC Hershey T. Longitudinal hearing loss in Wolfram syndrome. Orphanet J Rare Dis (2018) 13(102). doi: 10.1186/s13023-018-0852-0 PMC6020390 29945639

[52] Sun F Chai S Yu K Quan X Yang Z Wu S. Gastrointestinal adverse events of glucagon-like peptide-1 receptor agonists in patients with type 2 diabetes: A systematic review and network meta-analysis. Diabetes Technol Ther (2015) 17(1):35–42. doi: 10.1089/dia.2014.0188 25375397 PMC4290796

[53] Frias JP Bastyr EJ Vignati L Tschöp MH Schmitt C Owen K. The sustained effects of a dual GIP/GLP-1 receptor agonist, NNC0090-2746, in patients with type 2 diabetes. Cell Metab (2017) 26(2):343–352.e2. doi: 10.1016/j.cmet.2017.07.011 28768173

[54] Frias JP Nauck MA Van J Kutner ME Cui X Benson C. Efficacy and safety of LY3298176, a novel dual GIP and GLP-1 receptor agonist, in patients with type 2 diabetes: a randomised, placebo-controlled and active comparator-controlled phase 2 trial. Lancet (2018) 392(10160):2180–93. doi: 10.1016/S0140-6736(18)32260-8 30293770

[55] Knop FK Vilsbøll T Højberg P V. Larsen S Madsbad S Holst JJ. The insulinotropic effect of GIP is impaired in patients with chronic pancreatitis and secondary diabetes mellitus as compared to patients with chronic pancreatitis and normal glucose tolerance. Regul Pept (2007) 144(1–3):123–30. doi: 10.1016/j.regpep.2007.07.002 17692937

[56] El K Gray SM Capozzi ME Knuth ER Jin E Svendsen B. GIP mediates the incretin effect and glucose tolerance by dual actions on α cells and β cells. Sci Adv (2021) 7(11):1–11. doi: 10.1126/SCIADV.ABF1948 PMC7954443 33712466

[57] Willard FS Douros JD Gabe MBN Showalter AD Wainscott DB Suter TM. Tirzepatide is an imbalanced and biased dual GIP and GLP-1 receptor agonist. JCI Insight (2020) 5(17):1–16. doi: 10.1172/jci.insight.140532 PMC7526454 32730231

[58] Novikoff A O’Brien SL Bernecker M Grandl G Kleinert M Knerr PJ. Spatiotemporal GLP-1 and GIP receptor signaling and trafficking/recycling dynamics induced by selected receptor mono- and dual-agonists. Mol Metab (2021) 49(February):101181. doi: 10.1016/j.molmet.2021.101181 33556643 PMC7921015

[59] Jones B Buenaventura T Kanda N Chabosseau P Owen BM Scott R. Targeting GLP-1 receptor trafficking to improve agonist efficacy. Nat Commun (2018) 9(1):1602. doi: 10.1038/s41467-018-03941-2 29686402 PMC5913239

[60] Fonseca SG Fukuma M Lipson KL Nguyen LX Allen JR Oka Y. WFS1 is a novel component of the unfolded protein response and maintains homeostasis of the endoplasmic reticulum in pancreatic β-cells. J Biol Chem (2005) 280(47):39609–15. doi: 10.1074/jbc.M507426200 16195229

[61] Irwin N McClean PL Cassidy RS Finbarr PMO Green BD Gault VA. Comparison of the anti-diabetic effects of GIP- and GLP-1-receptor activation in obese diabetic (ob/ob) mice: studies with DPP IV resistant N-AcGIP and exendin(1–39)amide. Diabetes Metab Res Rev (2007) 23:572–9. doi: 10.1002/dmrr.729 17315241

[62] Pandiri AR. Overview of exocrine pancreatic pathobiology. Toxicol Pathol (2013) 42(1):207–16. doi: 10.1177/0192623313509907 PMC4360889 24190915

[63] Chandra R Liddle RA. Regulation of pancreatic secretion. Pancreapedia (2015). doi: 10.3998/panc.2015.38

[64] Morikawa S Blacher L Onwumere C Urano F. Loss of function of WFS1 causes ER stress-mediated inflammation in pancreatic beta-cells. Front Endocrinol (Lausanne) (2022) 13:849204(March). doi: 10.3389/fendo.2022.849204 35399956 PMC8990750

[65] Campbell SA Golec DP Hubert M Johnson J Salamon N Barr A. Human islets contain a subpopulation of glucagon-like peptide-1 secreting α cells that is increased in type 2 diabetes. Mol Metab (2020) 39:101014. doi: 10.1016/j.molmet.2020.101014 32413586 PMC7260680

[66] Vasu S Moffett RC Thorens B Flatt PR. Role of endogenous GLP-1 and GIP in beta cell compensatory responses to insulin resistance and cellular stress. PloS One (2014) 9(6). doi: 10.1371/journal.pone.0101005 PMC4072716 24967820

[67] Eiberg H Hansen L Kjer B Hansen T Pedersen O Bille M. Autosomal dominant optic atrophy associated with hearing impairment and impaired glucose regulation caused by a missense mutation in the WFS1 gene. J Med Genet (2006) 43:435–40. doi: 10.1136/jmg.2005.034892 PMC2649014 16648378

[68] Hansen AMK Bödvarsdottir TB Nordestgaard DNE Heller RS Gotfredsen CF Maedler K. Upregulation of alpha cell glucagon-like peptide 1 (GLP-1) in Psammomys obesus—an adaptive response to hyperglycaemia? Diabetologia (2011) 54(6):1379–87. doi: 10.1007/s00125-011-2080-1 21347622

[69] Ellingsgaard H Hauselmann I Schuler B Habib AM Baggio LL Meier DT. Interleukin-6 enhances insulin secretion by increasing glucagon-like peptide-1 secretion from L cells and alpha cells. Nat Med (2011) 17(11):1481–9. doi: 10.1038/nm.2513 PMC4286294 22037645

[70] Furth-Lavi J Hija A Tornovsky-Babeay S Mazouz A Dahan T Stolovich-Rain M. Glycemic control releases regenerative potential of pancreatic beta cells blocked by severe hyperglycemia. Cell Rep (2022) 41(9):111719. doi: 10.1016/j.celrep.2022.111719 36450253 PMC9789023

[71] Ben-Othman N Vieira A Courtney M Record F Gjernes E Avolio F. Long-term GABA administration induces alpha cell-mediated beta-like cell neogenesis. Cell (2017) 168(1–2):73–85.e11. doi: 10.1016/j.cell.2016.11.002 27916274

[72] Wang C Mao R Van De Casteele M Pipeleers D Ling Z. Glucagon-like peptide-1 stimulates GABA formation by pancreatic β-cells at the level of glutamate decarboxylase. Am J Physiol - Endocrinol Metab (2007) 292(4):1201–6. doi: 10.1152/ajpendo.00459.2006 17190904

[73] Bailey SJ Ravier MA Rutter GA. Glucose-dependent regulation of γ-aminobutyric acid (GABA A) receptor expression in mouse pancreatic islet α-cells. Diabetes (2007) 56(2):320–7. doi: 10.2337/db06-0712 17259375

[74] Bansal P Wang S Liu S Xiang YY Lu WY Wang Q. GABA coordinates with insulin in regulating secretory function in pancreatic INS-1 β-Cells. PloS One (2011) 6(10). doi: 10.1371/journal.pone.0026225 PMC3198728 22031825

[75] Soltani N Qiu H Aleksic M Glinka Y Zhao F Liu R. GABA exerts protective and regenerative effects on islet beta cells and reverses diabetes. Proc Natl Acad Sci U. S. A. (2011) 108(28):11692–7. doi: 10.1073/pnas.1102715108 PMC3136292 21709230

[76] Tian J Chau C Hales TG Kaufman DL. GABA(A) receptors mediate inhibition of T cell responses. J Neuroimmunol (1999) 96(1):21–8. doi: 10.1016/S0165-5728(98)00264-1 10227421

[77] Nakagawa T Yokozawa T Kim HJ Shibahara N. Protective effects of .GAMMA.-aminobutyric acid in rats with streptozotocin-induced diabetes. J Nutr Sci Vitaminol (Tokyo) (2005) 51(4):278–82. doi: 10.3177/jnsv.51.278 16262002

[78] Qiu Y Zhao Z Klindt D Kautzky M Szatko KP Schaeffel F. Natural environment statistics in the upper and lower visual field are reflected in mouse retinal specializations. Curr Biol (2021) 31(15):3233–3247.e6. doi: 10.1016/j.cub.2021.05.017 34107304

[79] Rossi G Ordazzo G Vanni NN Castoldi V Iannielli A Di Silvestre D. MCT1-dependent energetic failure and neuroinflammation underlie optic nerve degeneration in Wolfram syndrome mice. Elife (2023) 12:e81779. doi: 10.7554/eLife.81779 36645345 PMC9891717

[80] Barrett TG Bundey SE. Wolfram (DIDMOAD) syndrome. J Med Genet (1997) 34(10):838–41. doi: 10.1136/jmg.34.10.838 PMC1051091 9350817

[81] De Heredia ML Clèries R Nunes V. Genotypic classification of patients with Wolfram syndrome: Insights into the natural history of the disease and correlation with phenotype. Genet Med (2013) 15(7):497–506. doi: 10.1038/gim.2012.180 23429432

[82] Tirzepatide monotherapy in patients with wolfram syndrome type 1. Available at: https://clinicaltrials.gov/ct2/show/NCT05659368 (Accessed May 19, 2023).

[83] Alsalim W Lindgren O Ahrén B. Glucose-dependent insulinotropic polypeptide and glucagon-like peptide-1 secretion in humans: Characteristics and regulation. J Diabetes Investig (2023) 14(3):354–61. doi: 10.1111/jdi.13962 PMC9951578 36539382

[84] Zhang L-Y Jin Q-Q Hölscher C Li L. Glucagon-like peptide-1/glucose-dependent insulinotropic polypeptide dual receptor agonist DA-CH5 is superior to exendin-4 in protecting neurons in the 6-hydroxydopamine rat Parkinson model. Neural Regen Res (2021) 16(8):1660. doi: 10.4103/1673-5374.303045 33433498 PMC8323666

