# Supplementary figures and images for: Treatment with the dual-incretin agonist DA-CH5 demonstrates potent therapeutic effect in a rat model of Wolfram Syndrome
Source: Front Endocrinol (Lausanne). 2023 Oct 13;14:1234925. doi: 10.3389/fendo.2023.1234925 (PMC10611518; doi:10.3389/fendo.2023.1234925)

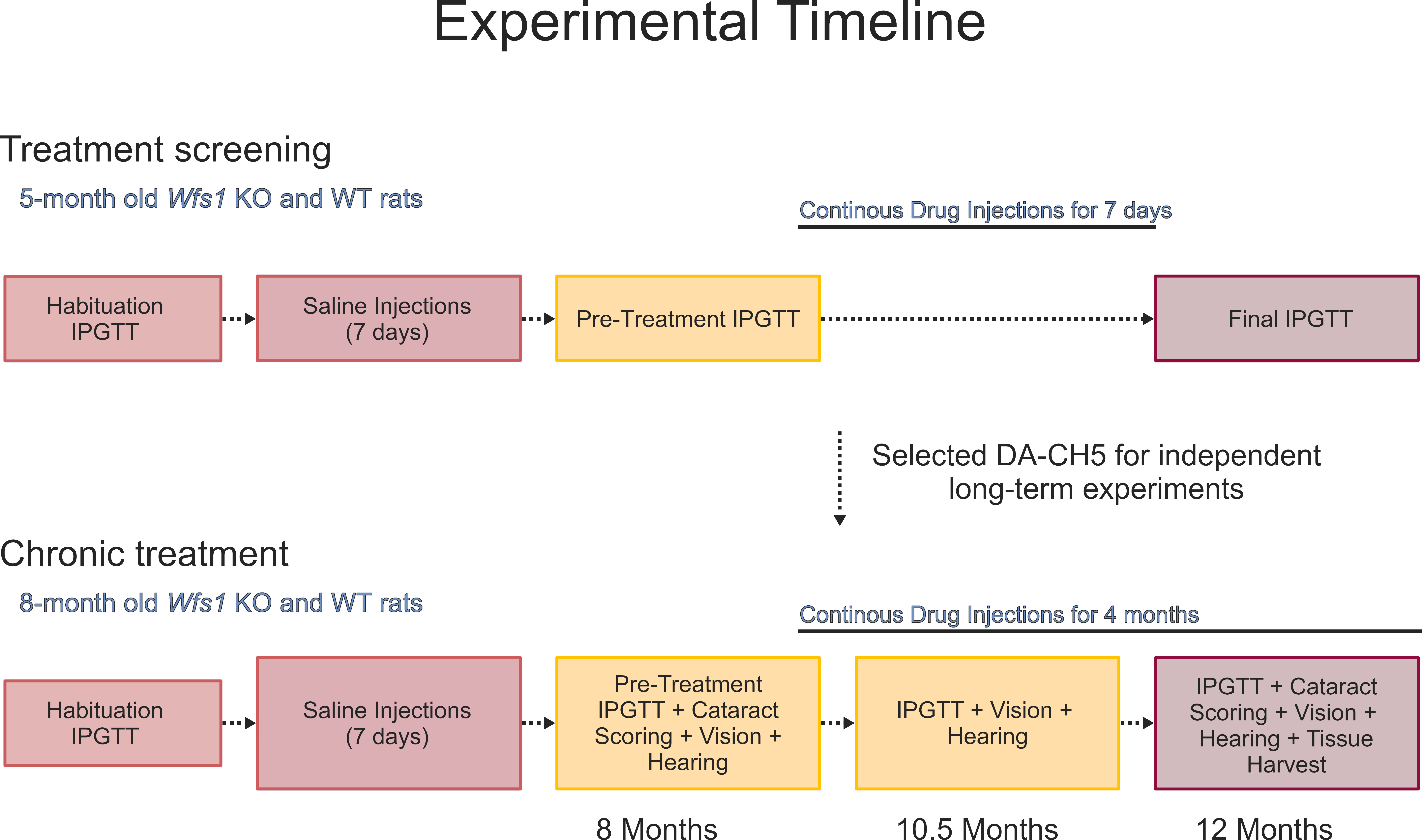

Supplement: Supplementary Figure 1 — Experimental timeline. Schematic representation of experimental protocols in acute and long-term treatment paradigms. [file Image_1.jpeg]

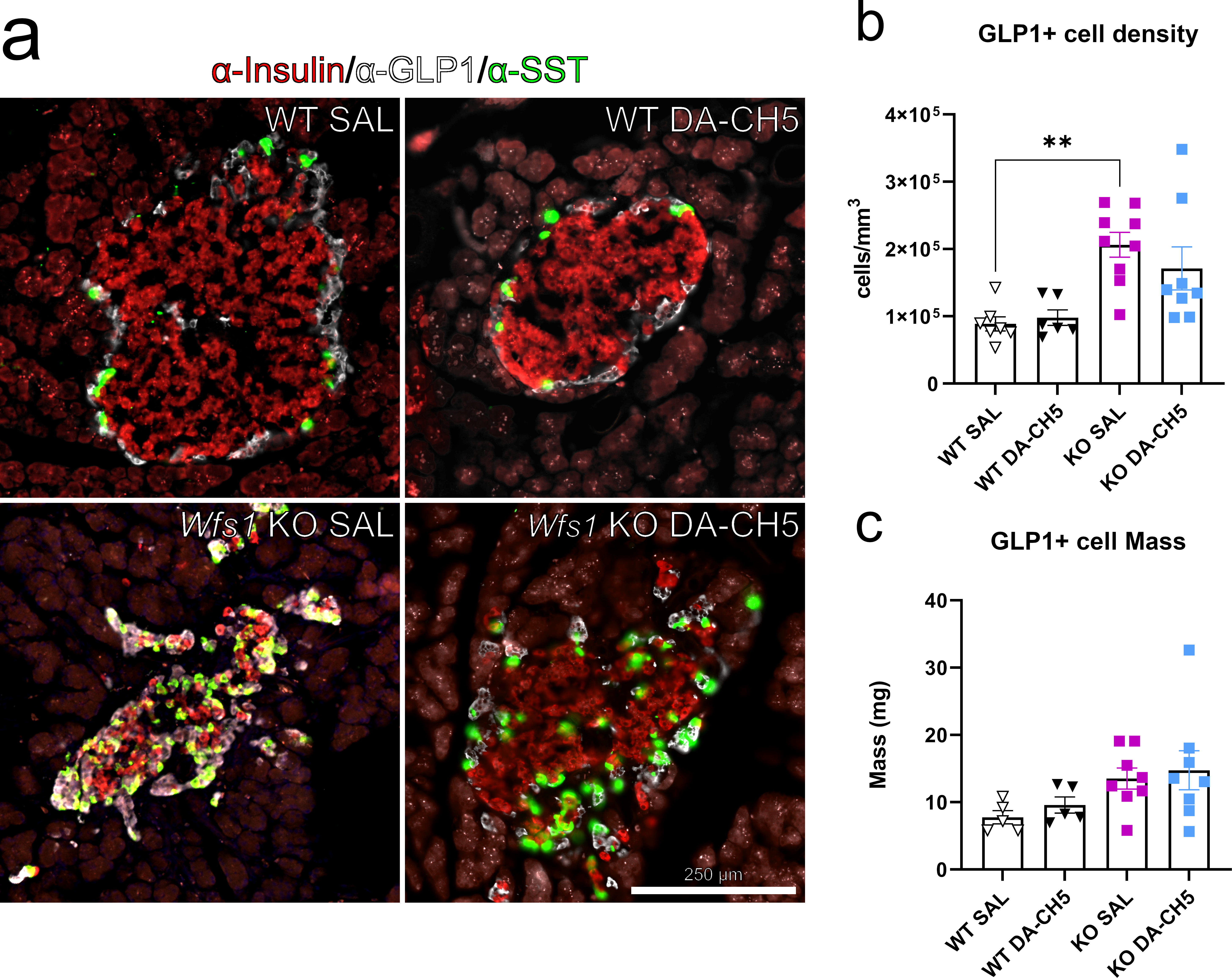

Supplement: Supplementary Figure 3 — Endocrine islet immunostaining for Glucagon-like Peptide 1. Eight-month-old wild-type (WT) and Wfs1 KO rats were continuously treated with either saline (SAL) or the dual-incretin agonist DA-CH5 (25 nmol/kg) for four months, following which pancreata were harvested and histologically examined. (A) Representative confocal images of endocrine islets immunostained for insulin (red), GLP-1 (grey), and somatostatin (SST, green). (B, C) GLP-1+ cell density and cell mass were quantified to evaluate between-group differences. Data are presented as the mean and standard error of the mean. Symbols represent individual mice. Two-way analysis of variance. * p < 0.05, ** p < 0.01, *** p < 0.001, **** p < 0.0001, (n = 5-8/group). Scale bar, 250 µm. [file Image_3.jpeg]

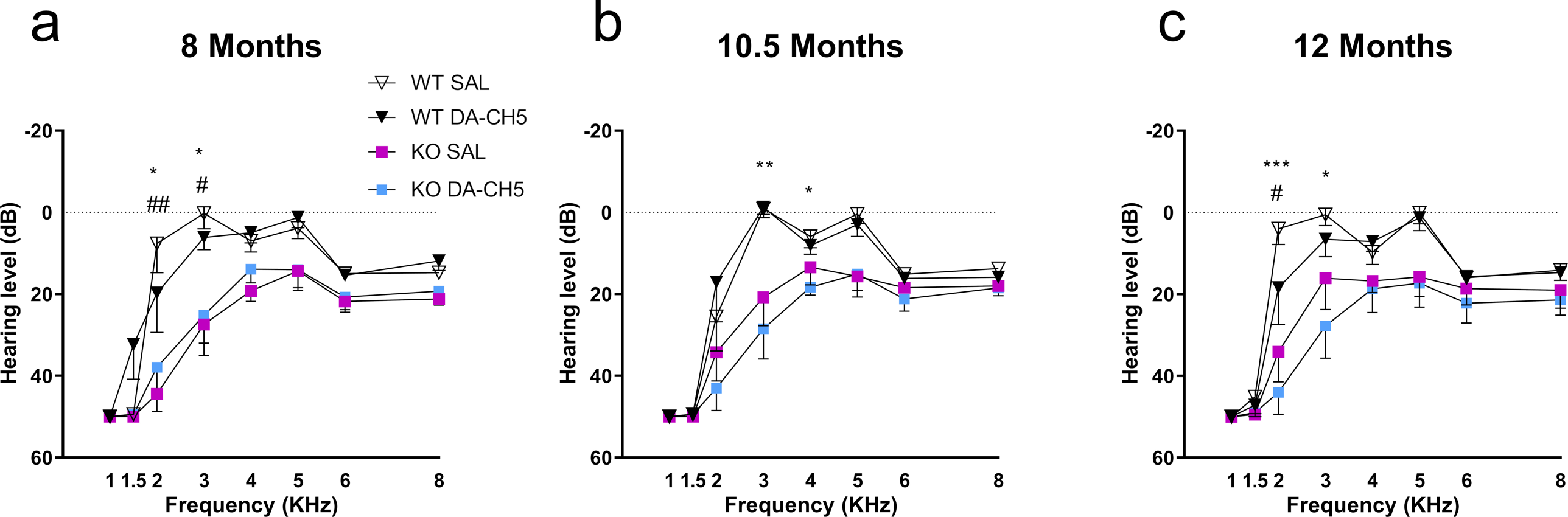

Supplement: Supplementary Figure 5 — Long-term dual-incretin agonist treatment does not mitigate hearing loss in Wfs1 KO rats. (A-C) Eight-month-old wild-type (WT) and Wfs1 KO rats were continuously treated with either saline (SAL) or the dual-incretin agonist DA-CH5 (25 nmol/kg) for four months. Cochlear hearing levels were estimated by otoacoustic emissions at (A) pre-treatment 8 months, (B) 10.5, and (C) 12 months of age. Data are presented as the mean and standard error of the mean. Two-way analysis of variance (n = 8-10/group). # WT SAL vs. KO SAL at p < 0.05, ## WT SAL vs. KO SAL at p < 0.01, ### WT SAL vs. KO SAL at p < 0.001. * WT SAL vs. KO DA-CH5 at p < 0.05, ** WT SAL vs. KO DA-CH5 at p < 0.01, *** WT SAL vs. KO DA-CH5 at p < 0.001. [file Image_5.jpg]
